# Development of a novel 3D-printed dynamic anthropomorphic thorax phantom for evaluation of four-dimensional computed tomography

**DOI:** 10.1016/j.phro.2024.100656

**Published:** 2024-10-20

**Authors:** Didier Lustermans, Roua Abdulrahim, Vicki Trier Taasti, Juliane Szkitsak, Evita Švėgždaitė, Sarina Clarkin, Brigitte Reniers, Frank Verhaegen, Gabriel Paiva Fonseca

**Affiliations:** a Department of Radiation Oncology (Maastro), GROW Research Institute for Oncology and Reproduction, Maastricht University Medical Centre+, Maastricht, The Netherlands; b Research Group NuTeC, Centre for Environmental Sciences, Hasselt University, Diepenbeek, Belgium; c Danish Centre for Particle Therapy, Aarhus University Hospital, Aarhus, Denmark; d Department of Radiation Oncology, Universitätsklinikum Erlangen, Friedrich-Alexander-Universität Erlangen-Nürnberg, Erlangen, Germany

**Keywords:** 4DCT, 3D-printing, 4D imaging phantom, 4DCT artifacts, Tumor motion, 4DCT quality assurance

## Abstract

**Background and purpose:**

In radiotherapy, the image quality of four-dimensional computed tomography (4DCT) is often degraded by artifacts resulting from breathing irregularities. Quality assurance mostly employ simplistic phantoms, not fully representing complexities and dynamics in patients. 3D-printing allows for design of highly customized phantoms. This study aims to validate the proof-of-concept of a realistic dynamic thorax phantom and its 4DCT application.

**Materials and methods:**

Using 3D-printing, a realistic thorax phantom was produced with tissue-equivalent materials for soft tissue, bone, and compressible lungs, including bronchi and tumors. Lung compression was facilitated by motors simulating customized breathing curves with an added platform for application of monitoring systems. The phantom contained three tumors which were assessed in terms of tumor motion amplitude. Three 4DCT sequences and repeated static images for different lung compression levels were acquired to evaluate the reproducibility. Moreover, more complex patient-specific breathing patterns with irregularities were simulated.

**Results:**

The phantom showed a reproducibility of ±0.2 mm and ±0.4 mm in all directions for static 3DCT images and 4DCT images, respectively. Furthermore, the tumor close to the diaphragm showed higher amplitudes in the inferior/superior direction (13.9 mm) than lesions higher in the lungs (8.1 mm) as observed in patients. The more complex breathing patterns demonstrated commonly seen 4DCT artifacts.

**Conclusion:**

This study developed a dynamic 3D-printed thorax phantom, which simulated customized breathing patterns. The phantom represented a realistic anatomy and 4DCT scanning of it could create realistic artifacts, making it beneficial for 4DCT quality assurance or protocol optimization.

## Highlights

• A novel 3D-printed thorax phantom was evaluated containing compressible lungs.
• It was tested for the application of four dimensional computed tomography.
• The phantom demonstrated a reproducibility of ±0.2 mm in tumor amplitudes.
• Patient-specific breathing patterns can be simulated and used for optimization.

## 1 Introduction

Four-dimensional computed tomography (4DCT) has revolutionized radiotherapy by allowing visualization and tracking of movements in tumors and organs-at-risk (OARs) [1], [2], [3], leading to more accurate treatment procedures [4], [5]. However, multiple studies have reported 4DCT acquisitions containing image artifacts [6], [7], [8], with at least one artifact in 90 % of the data [8]. These artifacts and the reduced image quality can negatively impact the 4DCT radiotherapy workflow [6], [9] in terms of contouring errors [10], [11] and lowered dose calculation accuracy [12], [13].

Severe motion artifacts often arise due to irregular breathing [6], [14], [15]. A European multicenter 4DCT quality-assurance (QA) study performed phantom tests at eleven institutions using one phantom but a range of different CT scanners [16]. This study suggested further improvements are needed in 4DCT imaging as large target volume differences (up to 16 %) were found. In conventional 4DCT, an irregular breathing pattern can lead to insufficient projection data coverage. Therefore, new 4DCT solutions have been developed to better synchronize the acquisition with the breathing signal [14], [15], [17], [18], [19]. This solution was evaluated in another multicenter 4DCT QA study [2], and showed a higher accuracy (CT number accuracy, volume deviation and amplitude deviation) with more irregular cases staying within the clinical threshold provided by the Canadian Partnership for Quality Radiotherapy (CPQR) [20]. In most studies, image quality and quantitative information measurements are performed using phantoms. However, the limited availability of dynamic phantoms presents a major limitation [2]. The most used phantom consists of a static structure with a cylinder moved by a piston, simulating breathing patterns [2], [17], [18]. However, this phantom does not realistically represent the complexities and lung deformation observed in patients. Non-commercial lung phantoms have been manufactured by combining multiple materials [21], [22], [23], [24].

Recently, three-dimensional (3D) printing has been introduced in radiotherapy, allowing for increased customization, and led to multiple novel applications [25], [26], [27], [28], [29]. This is also applied to patient-specific phantom manufacturing for imaging and dosimetry, including regions such as the head [30], [31] and thorax [32], [33], [34], [35], or to customize parts within commercial phantoms [18]. One of the most used 3D-printing methods is to model the phantom based on the CT numbers of a CT image. However, caution is needed if the phantom is used for quantitative imaging or dosimetry, as some materials provide approximately accurate CT numbers, but demonstrate differences in physical properties needed for accurate dosimetry [36], [37], [38]. In particular, materials with metal suspended in plastic were advocated to mimic bone [39], but differences were found in the effective atomic number (Z_eff_) and therefore a custom PolyLactide (PLA) filament combined with Calcium (PLA + Ca) was proposed for simulating bone [36]. Additionally, many studies have tried to achieve a low mass density for lung tissue by finetuning the infill pattern (small air inclusions) [32], [33], [40], [41], [42]. Furthermore, until recently, 3D-printed lung phantoms were mostly static and could not account for complex breathing motion as seen in patients.

This study investigated the manufacturing of a thorax phantom with dynamic lungs applicable for 4DCT. The aim of the study was two-fold. Firstly, a proof-of-concept to develop an anthropomorphic thorax phantom containing materials to mimic bone, lung and soft tissue for imaging and dosimetry. Secondly, to evaluate the phantom in 4DCT applications in terms of simulated tumor motion and reproducibility.

## 2 Material and methods

### 2.1 Phantom

**Fig. 1:**
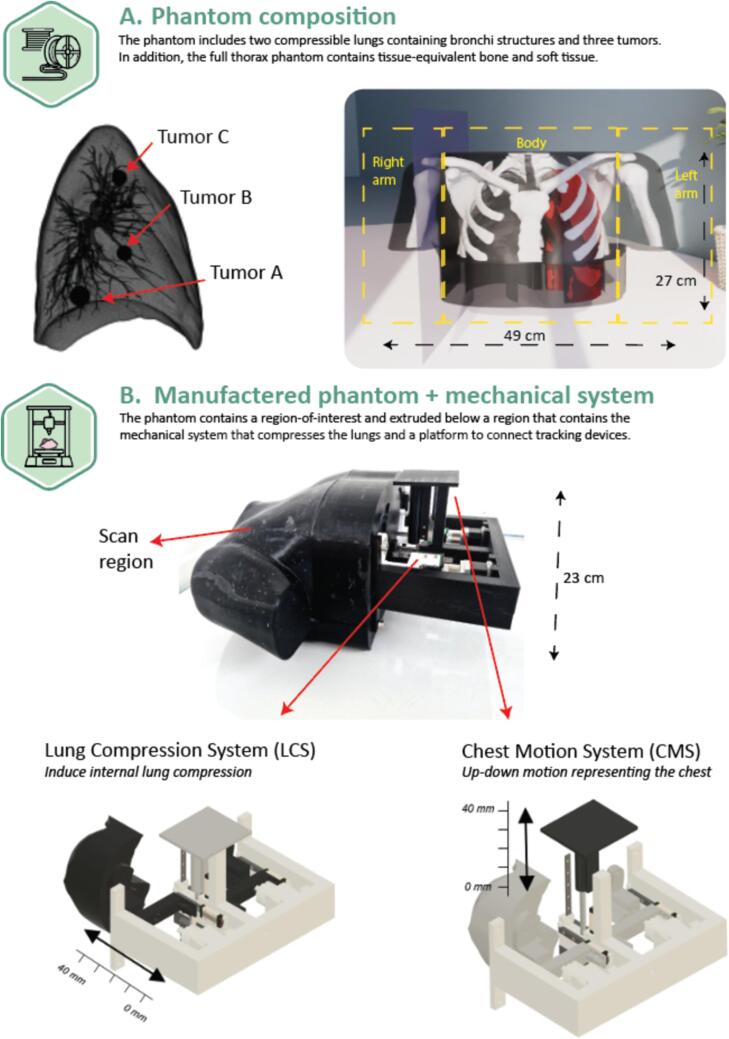
A visual representation of the composition of the manufactured thorax phantom (A) and an image of the printed version, together with a model explaining the mechanical systems (B). A) The phantom consists of soft tissue, bone and two flexible lungs. The latter contains internal structures such as bronchi and three tumors placed in different lung segments. The phantom was printed in three separate pieces (left arm, body, right arm) due to printer dimension restrictions. These three parts were combined after printing. The dimension of the phantom is 27 × 49 × 23 cm (l × w × h). B) At the bottom of the phantom, two mechanical systems are connected, the lung compression system (LSC) and chest motion system (CMS). Both systems have a range up to 40 mm, but the actual degree of compression is limited by the compressibility of the lungs. The LCS is connected to a solid block for internal compression of the lungs by two motors. The CMS consists of a motor to simulate 1-dimensional chest motion and allows for placement of a respiratory gating device.

An anthropomorphic 4DCT thorax phantom was manufactured with fused deposition modeling (FDM) 3D-printing using tissue-equivalent printing materials (for CT X-ray imaging) that represented soft tissue, bone and lung tissue. The lungs were deformable and compressible with an internal structure containing bronchi and tumors (Fig. 1).

The lung compression was performed by an in-house developed electronic lung compression system (LCS) that allowed for simulation of realistic breathing motion and ensured the lung returned to its initial position. A second system, called the chest movement system (CMS), was added to introduce 1D anterior-posterior rigid motion of a platform simulating the chest wall. To address potential imaging artifacts caused by metal, the region near the diaphragm was printed as a solid plastic (PLA) object, ensuring the metal components were positioned at a sufficient distance from the region-of-interest (ROI) being the lungs.

The LCS was equipped with an individual motor for each lung, employed to simulate lung compression and tumor motion. These motors, with an accuracy ± 0.1 mm, were connected to solid plastic blocks fixed to the lungs and replicated breathing motion with a prescribed amplitude. Meanwhile, the CMS featured a single motor to replicate chest wall motion. A solid block was attached to this motor, providing a platform for the integration of respiratory gating devices (Fig. 1). Software was developed to move each motor independently, based on mathematical functions (e.g. sine functions) or patient breathing patterns.

### 2.2 Material and model selection

The 4DCT thorax anatomy was extracted from the extended cardiac-torso (XCAT) [43] mathematical model. Due to printer dimension restrictions, the model was divided into three body sections as seen in Fig. 1. The lung model included a realistic bronchial tree and contained three spherically shaped solid lesions in the lower (volume of 3.49 cm^3^; tumor A), middle (1.47 cm^3^; tumor B), and upper (1.47 cm^3^; tumor C) section of the left lung.

Materials were selected based on tissue-equivalence by extracting the physical properties (e.g. Z_eff_ [44]) as done in a previous study [36], using a dual-energy CT scanner (DECT; Siemens SOMATOM Definition Drive) and the syngo.via DECT package (Siemens Healthineers, Forchheim, Germany). Black PLA (Real Filament, Almere, The Netherlands) showed a Z_eff_ of 6.9 ± 0.4, with the closest resemblance to commercial high-equivalence (HE) adipose tissue (6.6 ± 0.7) and HE solid water (7.4 ± 0.5; Gammex AED phantom, Sun Nuclear, Middleton, WI, USA). This was chosen to represent soft tissue while the custom PLA + Ca filament (Z_eff_ of 11.1 ± 0.1) [36] was chosen to represent bone. Furthermore, Thermoplastic Polyrurethane (TPU; Recreus Industries, Elda, Spain) was used to approximate lung tissue (Z_eff_ of 10.1 ± 0.3).

### 2.3 3D-printing

The soft tissue and bone anatomical structures were fabricated with a dual extruder process to deposit the filaments in the same print, whereas the lungs were manufactured with a single direct extruder process. This type of nozzle decreases the distance between deposition and the nozzle gears, leading to less clogs.

The body structure was printed with filaments that used different structural configurations (Fig. S1 in the Supplementary Material (SM)), with PLA using a line pattern and PLA + Ca a gyroid structure. With the aim to simulate the anatomy of compact bone and spongy bone, the bones (PLA + Ca) were printed with four consecutive shells (simulating compact bone) and a printing flow of 110 % (amount of material extruded when printing), whereas the internal structure (simulating spongy bone) was printed with a flow of 85 %. This was intended to obtain a higher density and consequently increasing the CT number in the shells. The lungs were printed with a low speed of 22.5 mm/s and nozzle temperature, leading to improved printing conditions. A small nozzle size was used for lung printing to ensure a low density and a homogeneous structure as seen on a CT image. To make them compressible in the superior-inferior direction, they were printed as demonstrated in Fig. S1. Printing settings and materials are listed in Table 1 and more details can be found in SM.

**Table 1:** List of the used 3D-printing filaments, the filament characteristics (including effective atomic number, Z_eff_, estimated by dual-energy CT), and the printer settings. * Flow settings for the lung tissue is an approximation, as this setting was changed during printing by visually inspecting the quality of the print and a decision was made to increase or decrease the flow depending on the material deposition.

	**Soft tissue**	**Bone**	**Lung tissue**
Material Type	PLA – Black	Bone Filament	TPU – Red 70A
Brand	Real Filaments	Colorfab	Recreus Industries, FilaFlex
Printing Temperature [°C]	210	210	240
Infill Density [%]	100	100	24
Flow [%]	85	85	*∼120
First layer flow [%]	85	85	145
Nozzle diameter [mm]	0.6	0.6	0.25
Layer height [mm]	0.4	0.4	0.19
Line Width [mm]	0.7	0.7	0.24
Infill Pattern	Lines	Gyroid	Gyroid
Shells	1	4	0
Z_eff_	6.9 ± 0.4	11.1 ± 0.1	10.1 ± 0.3

### 2.4 Image acquisition

4DCT images of the manufactured phantom were acquired with a SOMATOM Definition Drive CT scanner (Siemens Healthineers, Forchheim, Germany) with a tube voltage of 120 kVp. The Anzai chest motion belt (Anzai Medical Co. Ltd, Tokyo, Japan) was used to record the breathing signal. The 4DCT was reconstructed into eight respiratory phases using amplitude-binning (25 % phase increments), as well as an average 4DCT. The scan parameters for the 4DCT were chosen based on clinical practice including a pitch of 0.14, a field-of-view of 500 mm, and a CTDI_vol,32 cm_ of 22 mGy. Reconstruction was performed with a quantitative kernel (Qr40) and iterative reconstruction (ADMIRE level 3), slice thickness of 3 mm, and beam hardening correction (iBHC) for bone. In addition, static 3DCT images (120 kVp) were acquired with different compression levels of the lungs (0 mm, 10 mm, and 20 mm). Reconstruction was performed with CT parameters equal to the 4DCT acquisition, except with a decrease in slice thickness to 0.5 mm.

### 2.5 4DCT evaluation

The 4DCT application was assessed by evaluating the tumor position in different phases of two simulated regular sinusoidal breathing curves, to focus on the phantom accuracy rather than the 4DCT scanner performance. An amplitude of 14 mm (referred to as Reg14mm) and 20 mm (referred to as Reg20mm), both with a period of 4 s, was used based on another study [45], see Fig. S2 in SM. These amplitudes controlled the compression of the lungs and not directly the tumor motion amplitude. Therefore, tumors were segmented in all phases of the 4DCT by thresholding (CT number > -200 Hounsfield Unit [HU]), and the lesion position was quantified by the center-of-mass for all three tumors to assess the behavior in different lobe segments.

To validate the phantom motion reproducibility, repetition tests were performed by applying the three compressions in sequence, and repeating this sequence five times, to check whether the phantom returned to the same position. These tests were initially performed on static 3DCT images, to rule out differences due to the 4DCT amplitude-binning. The tumor amplitude (inferior/superior [I-S], anterior/posterior [A-P] and lateral [LAT]) was quantified in each scan. Moreover, a 4DCT acquisition of the Reg20mm was repeated three times as non-consecutive measurements and quantitatively assessed the tumor motion amplitude in I-S direction. In addition to the simple sinusoidal curves, two more complex patterns were simulated with large amplitude irregularities (Pat1) and a breathing pause (Pat2), see Fig. S3 in SM, and qualitatively evaluated.

## 3 Results

**Fig. 2:**
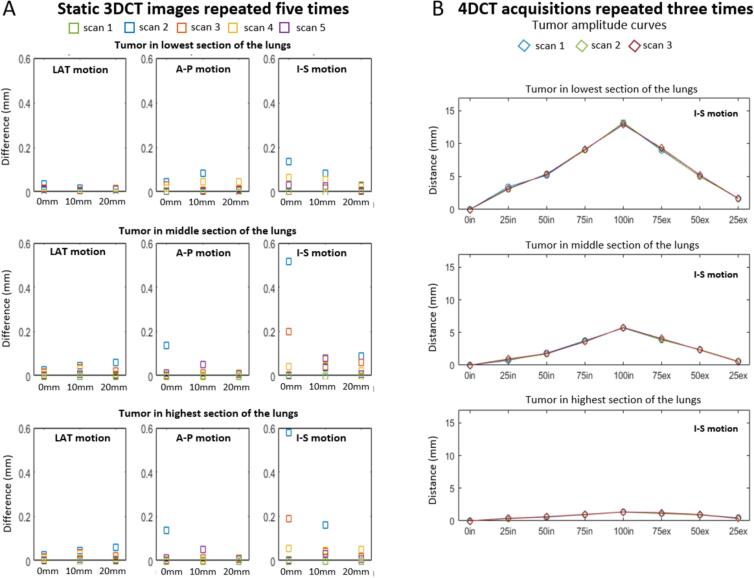
A) The tumor position difference in three directions (LAT, A-P, and I-S) compared to an initial static 3DCT image. Each color represents a new cycle of compressing the lung (0 mm, 10 mm, and 20 mm). The motion difference is shown for each tumor (placed in the low, middle, and upper section of the lung). B) Graphs indicating the tumor position in each 4DCT phase for I-S motion of three repeated scans. The lines are not a fit, but just demonstrative to connect the points.

For the repeated 3DCTs of the static phantom, all three tumors consistently had a position difference less than 0.2 mm in all directions for all three levels of lung compression, with one exception of 0.52 mm for the lesion in the highest section of the lung (Fig. 2A). In addition, the position difference between the 0 mm and 10 mm or 20 mm was evaluated, see Fig. S5 in SM. In the LAT and A-P directions almost no tumor displacement was seen. The displacement in the I-S direction was naturally much larger as this was the direction of the lung compression, but the positions for each compression level in the different repetitions were almost overlaying each other, except for in one scan where a relatively small deviation was seen for tumor C.

The lesion position difference in each phase of three repeated 4DCT scans was small (Fig. 2B). Again, the LAT and A-P direction showed a small displacement, whereas the I-S direction showed displacements up to 13.2 mm, 5.8 mm, and 1.4 mm for the low, middle, and upper positioned tumor, respectively. The largest difference between the three scans was 0.4 mm in the I-S direction.

**Fig. 3:**
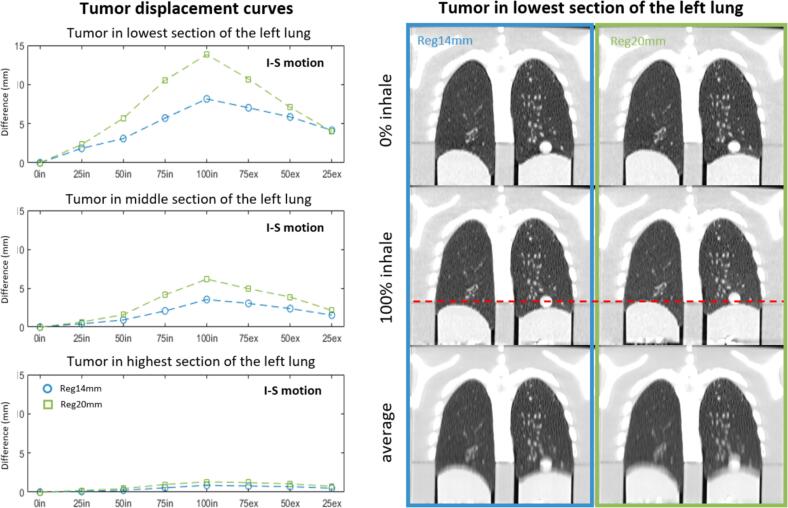
Left) Three graphs demonstrating the inferior-superior (I-S) tumor displacement of each lesion based on the eight phases of the 4DCT, for the two regular breathing patterns, the 14 mm lung compression (Reg14mm; blue) and the 20 mm lung compression (Reg20mm; green). Note: The dashed lines in the graphs are not a fit, but connection between the points. Right) A visual representation of the 0 % inhale and 100 % inhale phase as well as the average 4DCT for both curves. The red dashed line indicates the difference in lesion position for the 100 % inspiration between the Reg14mm and Reg20mm. Abbreviations: in – inspiration, ex – expiration, numbers – percentage of breathing phase. (For interpretation of the references to color in this figure legend, the reader is referred to the web version of this article.)

The regular breathing pattern simulation was tracked correctly by the 4DCT. The tumor amplitudes and a visualization of an average 4DCT and the two extreme phases are shown in Figs. 3 and S4 in SM. As the system controlled the compression and not directly the lesion motion, lesion amplitudes were smaller than 14 or 20 mm. The I-S displacements of the tumor in the lower lung demonstrated a maximum amplitude of 8.1 mm for the Reg14mm and 13.9 mm for the Reg20mm. When looking at tumor B, the difference between the minimum and maximum inspiration was 3.5 mm and 6.3 mm for the Reg14mm and Reg20mm curves, respectively. This demonstrates a reduction in displacement when the tumor is located higher in the lung, with the upper tumor reaching a maximum of 1.3 mm in I-S displacement.

**Fig. 4:**
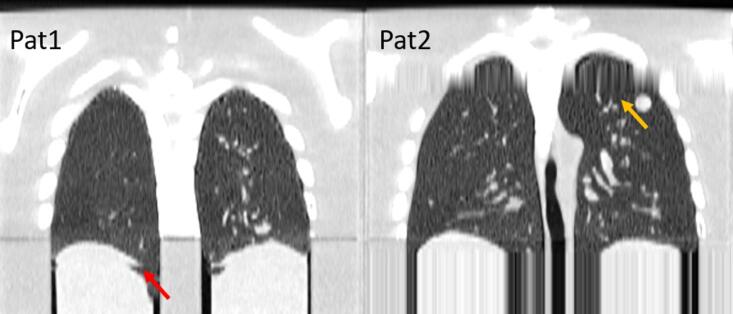
Images of more complex breathing patterns with irregularities that contain large amplitude changes (Pat1) and a breathing pause (Pat2). The red arrow indicates a double-structure artifact and the orange arrow an interpolation artifact arising from the irregularities. (For interpretation of the references to color in this figure legend, the reader is referred to the web version of this article.)

More complex breathing patterns with large irregularities were simulated resulting in artifacts, as seen in Fig. 4. The data of Pat1 demonstrated double-structure artifacts. The image of Pat2 showed artifacts at multiple regions due to interpolation artifact, limiting the image quality.

## 4 Discussion

This study showed a proof-of-concept of manufacturing a geometrically realistic and tissue-equivalent thorax phantom with compressible lungs for the application in 4DCT. It demonstrated one of the first 3D-printed flexible lungs, which allows for patient-specific customization and a reproducibility of ± 0.2 mm in tumor position. Additionally, it demonstrated the application in 4DCT by simulating simplistic breathing curves and more advanced patient-specific curves, as this could be important to evaluate artifact mitigation and image improvement techniques.

3D-printing in radiotherapy has experienced substantial growth, with most studies focusing on phantom development based on CT numbers seen in patient images [31], [33]. Some studies have gone a step further, by optimizing 3D-printing algorithms to achieve precise attenuation profiles [46], [47], [48]. However, most studies do not include physical property evaluation, which is important for dose calculation in radiotherapy. This study showed a high resemblance in Z_eff_ of soft tissue and bone compared to tissue-equivalent materials. This allows for more realistic modeling of imaging artifacts such as beam hardening, gives similar behavior as human bodies for different CT energies, and could be employed for dosimetry. For the lungs, mechanical properties were prioritized over tissue-equivalency, but more materials should be studied.

Other 3D-printed lung phantoms mostly employed a PLA filament to replicate lungs [32], [33], [40], [41]. However, this material cannot be deformed, whereas the flexible lungs manufactured in this study allow for application in 4DCT, e.g., as a tool for QA. Nowadays commercially available phantoms, which often lack realism, are employed for QA. However, their high costs hampers the availability in clinics [2]. The phantom in this study has a lung geometry and structure which resembles a human thorax, while still allowing a controlled internal and external motion. The CMS facilitates the addition of a motion monitoring system as also seen in commercial phantoms for 4DCT. The costs of internal production are low compared to commercial devices, as they have additional costs due to certification, customer support. In addition, local regulations such as Medical Device Regulations [49] may require additional documentation.

For one repeated 3DCT scan, the phantom demonstrated an uncertainty of 0.52 mm in the upper lesion. This larger displacement was due to lung movement when returning to a too low initial position (Fig. S6 in SM), as the lungs are not fixated to the soft tissue. Therefore, a slightly higher zero-position is needed in future measurements, to keep the lungs connected to the superior part of the phantom cavity. These tests were performed on 3DCT images to avoid uncertainties in amplitude-based sorting. However, acquiring 4DCT images at different times still showed high accuracy in tumor position with a reproducibility of ±0.4 mm. Small uncertainties in the 4DCT measurements can also arise due to segmentation of the lower lesion, since the CT numbers for the compression block and the tumor were close. Moreover, a sort of hysteresis is seen for both curves in Fig. 3 which could arise from differences in resistance faced by the motors or “delayed” internal movement due to the flexible material. However, as there is a control over the movement, corrections could be implemented to compensate. Additionally, relatively simple sinusoidal breathing waves were used, and not a cos^6^-motion-function as seen in the literature [50], [51], [16], to focus more on the phantom motion reproducibility during imaging rather than the performance of the system.

Within the regular breathing curves it was demonstrated that a larger I-S displacement was achieved for the lower lesion, compared to the middle and upper lung sections. Multiple studies evaluated this tumor amplitude behavior in patients demonstrating large variations between patients, ranging from 1.5 mm (upper-lobe) to 25 mm (lower-lobe) for unfixed tumors [12], [45]. However, the degree of motion is dependent on multiple factors such as respiratory motion, tumor size/shape, lung elasticity and position in the lung. Typically, tumor amplitudes in patients are larger in the lower lobe compared to the upper lobe [45], comparable to the phantom data. These studies also demonstrate motion in A-P and LAT direction due to heartbeat, which was not seen in this phantom. However, the displacement (up to 2 mm [45]) was significantly lower than in I-S direction.

A disadvantage of the phantom is the rigid chest which makes the phantom less realistic, and it is not MR compatible. A possible MR compatible solution could be to use a pressure pump-system as done previously [52], [53], [54]. An advantage of the 3D-printed lungs is that changing printer settings or filament shore hardness could facilitate variations seen in patients. Therefore, different elasticities could be achieved ranging from average lung capacity to idiopathic pulmonary fibrosis patients, enabling patient-specific application. This includes manufacturing multiple lungs and exchange them depending on the required I-S tumor amplitude. The customization possibility offers a large benefit in patient-specific application compared to other 4DCT phantoms [52], [53], [54]. The lungs can also be designed to include films to evaluate dosimetry and a hollow trachea was added to potentially include optical fibers or small ionization chambers. Additionally, the lung mechanical properties should be further studied to translate the phantom compression input towards directly tumor motion amplitude and to simulate differential tumor motion by varying printer settings.

In addition, patient-specific breathing curves can be simulated in the phantom, to demonstrate more complex patterns. Pat1 showed double-structure artifacts that can arise from amplitude and period changes [19] due to misalignment in phase-sorting and is seen mostly in end-inspiration [6]. The interpolation artifacts seen in Pat2 indicate a lack of sufficient projection data for reconstruction [6], [19] and could be caused by the breathing pause in this breathing pattern. Generating and mimicking artifacts, found in patients [6], [14], [15], in a realistic phantom is essential for optimizing the 4DCT acquisition protocol. It opens possibilities to perform direct comparison with a patient-like geometry, but more reproducible and ethical, as no dose is deposited. As an example, it could be employed to compare different software/hardware, such as regular 4DCT compared to i4DCT [14], [17], [18], [19]. However, evaluating the scanner performance was out of the scope of this study.

In conclusion, this study demonstrated the feasibility of producing a dynamic thorax phantom with 3D-printing. It allowed for an application in 4DCT, where it could mimic image artifacts in a realistic anatomy. This could offer possibilities to use it as a tool for 4DCT QA or optimizing the 4DCT protocols and image quality.

## Declaration of competing interest

The authors declare that they have no known competing financial interests or personal relationships that could have appeared to influence the work reported in this paper.
