# Supplementary material for: Development of a novel 3D-printed dynamic anthropomorphic thorax phantom for evaluation of four-dimensional computed tomography
Source: Phys Imaging Radiat Oncol. 2024 Oct 20;32:100656. doi: 10.1016/j.phro.2024.100656 (PMC11546439; doi:10.1016/j.phro.2024.100656)
Supplement: Supplementary Data 1 [file mmc1.pdf]

Supplementary materials

This is the Supplementary Materials of the manuscript "*Development of a novel 3D-printed dynamic anthropomorphic thorax phantom for evaluation of four-dimensional computed tomography*".

Image processing and visualization for this study have been performed in an in-house developed open-source software, called A Medical Image-based Graphical platform – Python (AMIGOpy; https://www.amigo-medphys.com/).

S1. Material selection and 3D printing process

This section will describe the selection and 3D-printing process of the anthropomorphic thorax phantom in more detail.

In this study, dual-energy computed tomography (DECT) scans were used to explore the physical properties of various PolyLactide (PLA), Acrylonitrile Butadiene Styrene (ABS), and PolyEthylene Terefthalate Glyco (PETG) filaments to determine a material in close resemblance to solid water properties. Understanding the physical properties of the various 3D-printing filaments could improve the tissue-equivalence of the phantom to replicate the X-ray attenuation profile seen for real human tissues and make the phantom applicable to dosimetry.

The black PLA filament from Real Filaments (Real Filaments, Almere, The Netherlands) demonstrated an effective atomic number (Z_{eff}) of 6.7 ± 0.4 and a relative electron density (RED) of 1.04 ± 0.03. The latter can be adjusted slightly by adjusting the printer's settings (Fonseca et al., 2023). This showed the most comparable result in terms of Z_{eff} compared to HE adipose tissue and HE solid water of the Gammex Advanced Electron Density (AED) phantom (Sun Nuclear – A Mirion Medical company, Middleton, WI, USA). The PLA+Ca was similar to commercial tissue-equivalent CB30 and CB50 inserts (Z_{eff} = 10.6 and 12.6, respectively) (Fonseca et al., 2023). The PLA and PLA+Ca were therefore chosen to represent soft tissue and bone, respectively. Prior to manufacturing, in-depth assessments were conducted to select the appropriate printer settings, as they influence the material compositions. Three PLA blocks, 5 cm x 5 cm x 3.8 cm, were printed that incorporated a centrally positioned PLA+Ca cylinder with increasing diameter of the cylinder from 2.7 cm to 3.3 cm (top to center), with the largest diameter in the center of the block. Differences between the blocks were the material flow (amount of material extruded when printing), at 95%, 90%, and 85%, as this could influence the RED and CT number. These blocks were inserted into a custom-made cylindrical 3D-printed holder and scanned on a Siemens SOMATOM Drive CT scanner (Siemens Healthineers). The CT numbers for the three different material flows were determined, with a mean of 82±15 Hounsfield Units (HU), 35±13 HU, and 15±11 HU for PLA, and 451±15 HU, 406±14 HU, and 357±11 HU for PLA+Ca, respectively.

The soft tissue and bone anatomical structures were fabricated using a Raise3D Pro3 Plus printer (Raise3D, Irvine, CA, USA), equipped with a dual extruder to deposit the PLA and PLA+Ca filaments in the same print. The printer bed dimensions were 30 cm x 30 cm, precisely fitting the internal body part of the phantom. The 3D models were prepared using the IdeaMaker 3D printing software (Raise3D, Irvine, CA, USA). The soft tissue block was imported to the software and the bone material was added as modifier within the soft tissue. This means that the settings for both filaments stayed equal, except that some settings were changed for the bone (see Table 1 in the main text), such as number of shells and infill pattern (see Figure S1). It took around 2 days to print each arm, while it took one week to print the body region. To ensure that the printer would not clog, the nozzle was cleaned every two days during the printing period.

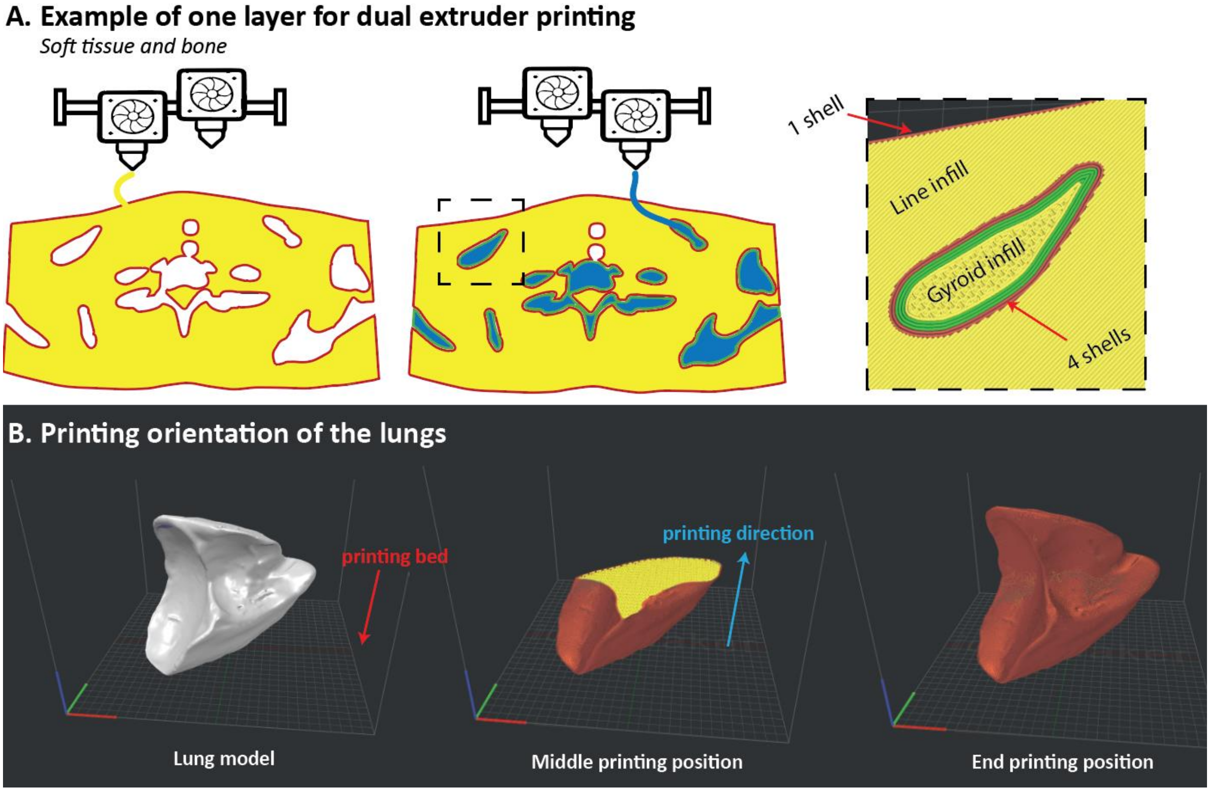

Figure S1*: A) Visualization of the printing process of the soft tissue and bone filament on a dual extruder printer. The zoom at the right (position marked with a dashed box in the middle figure) shows the differences in printing configurations and shells used for printing the soft tissue and bone materials. B) A representation of the printing direction of the lungs, demonstrating the model (left), a middle position during the print (middle), and the end result (right). The printing direction is important for the compressibility of the lungs, therefore the printing was performed along the Z-axis.*

The lungs were printed separately from the large body part, with flexible Thermoplastic Polyrurethane (TPU) to ensure compressibility. Various TPU materials were explored with different shore hardnesses, which is a measure of the resistance of a material to indentation, based on printing complexities and mechanical properties. Due to the mechanical properties, TPU FilaFlex 70A red (Recreus Industries, Elda, Spain) was chosen. More evaluation is needed to test the tissue-equivalence of the lungs.

The lungs were printed using the Ender v3 Pro (Creality 3D Technology, Shenzhen, China). This printer provided a print dimension of 22 cm x 22 cm and employed a direct extruder (Creality Sprite Pro, Creality 3D Technology, Shenzhen, China). The lungs were modeled based on a realistic geometry, with inclusion of a bronchial tree. These bronchi were added to the lungs in the same way as the bone were added to the soft tissue and used different printing settings. In addition, three solid masses were added to the lungs in different sections, the bottom (Tumor A; volume of 3.49 cm^3), the middle (Tumor B; 1.47 cm^3) and the upper section (Tumor C; 1.47 cm^3), to resemble tumors in the lung. For the compressibility of the lungs, it is important to print them in a certain direction, where they are oriented along its horizontal axis, see Figure S1.

Printing the lungs with flexible filaments is a challenge, and the manufacturer suggests a large nozzle size, but for a realistic outcome the lungs need a low density with a homogeneous look and therefore a small nozzle size is required. This can lead to multiple challenges. Firstly, a filament jam in the nozzle is possible when the distance between the gear and the nozzle is too far. Therefore, it was decided to use a direct extruder on the printer to decrease the distance between these components. Secondly, soft filament can increase the strain in the gears and pressure in the hotend resulting in clogging the system. Finding an optimal printing speed and temperature could help the printing process. When printing is performed too slowly, the filaments can already be softened within the extruder head and

not pushed out through the nozzle. Moreover, when the temperature is too low, the pressure will increase, and the nozzle could get clogged.

S2. Application of 4DCT

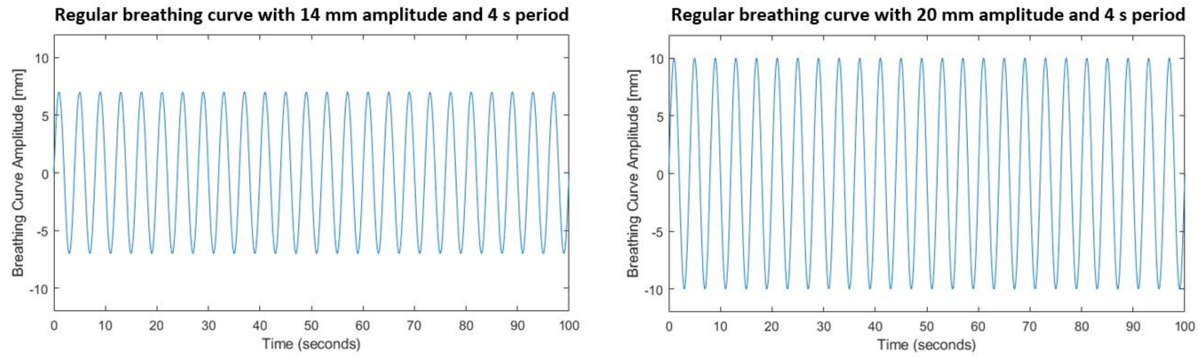

Figure S2: *Graphs showing the regular breathing curves simulated on the thorax phantom. The two curves are breathing curves with a period of 4 s with a simplistic sinusoidal shape (14 mm and 20 mm amplitude).*

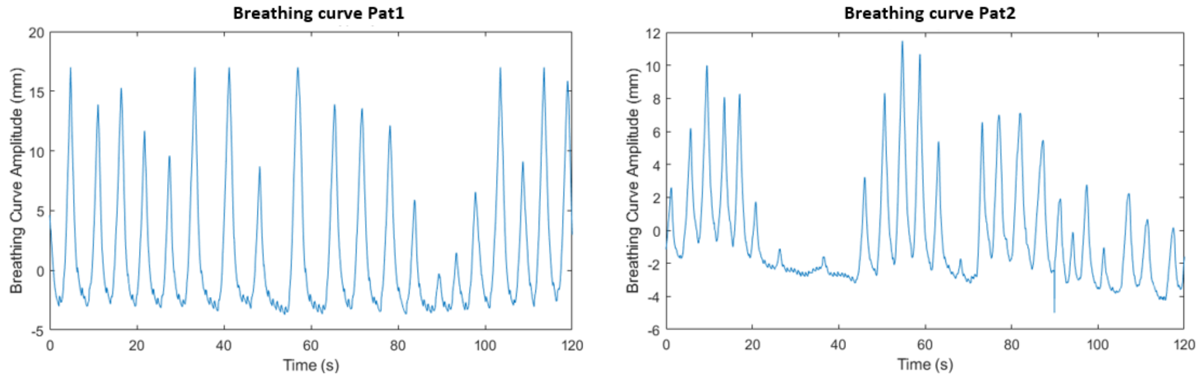

Figure S3: *Graphs showing the irregular breathing curves simulated on the thorax phantom. The Pat1 curve demonstrates a large irregularity in breathing amplitudes, whereas Pat2 shows a breathing pause.*

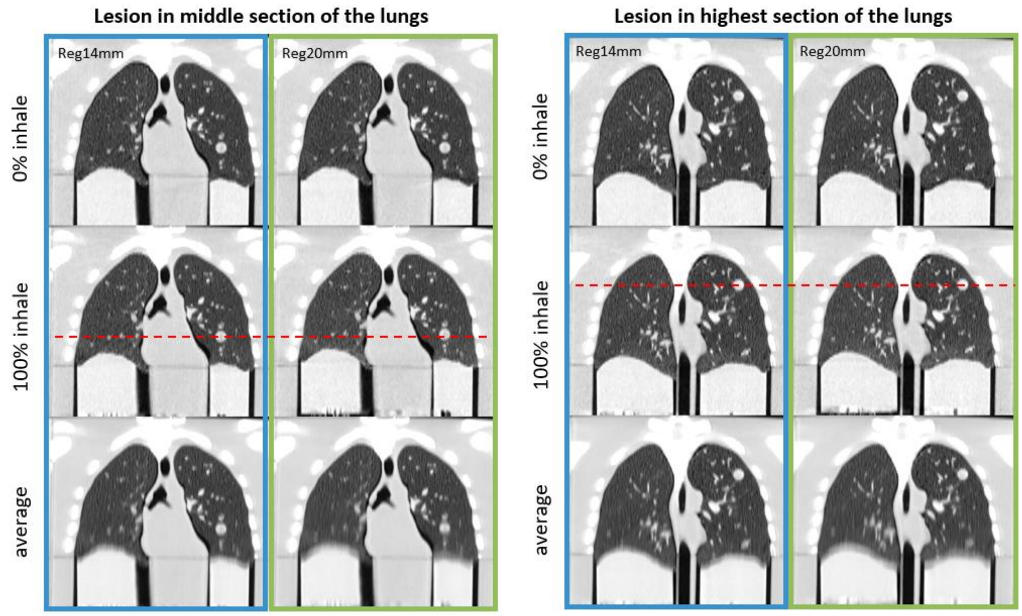

Figure S4: *A visual representation of the lesions in the middle and highest section of the lungs, for the 0% inhale and 100% inhale phase as well as the average 4DCT, for both the Reg14mm and the Reg20mm. The red dashed line indicates the difference in lesion position for the 100% inspiration between the Reg14mm and Reg20mm.*

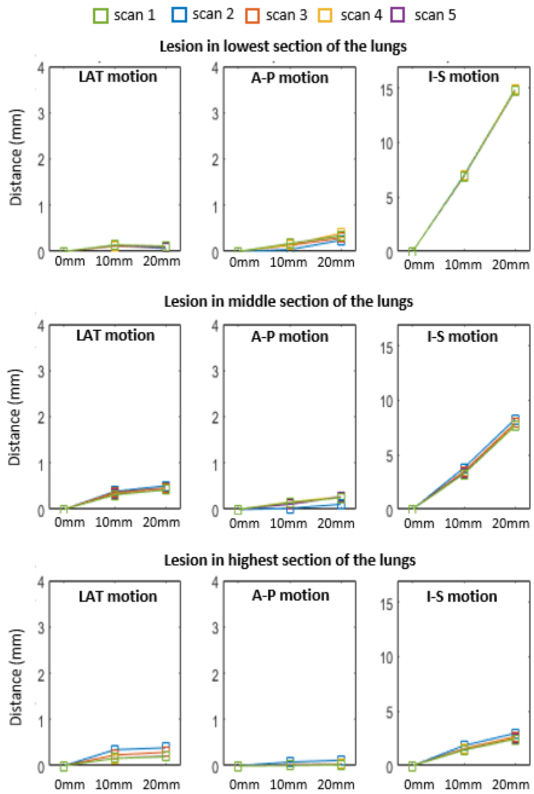

Figure S5: *The tumor position distance in three directions (LAT, A-P, and I-S), where each color represents a new cycle of compressing the lung (0 mm, 10 mm, and 20 mm) compared to the 0 mm compression position. Each lesion (low, middle, and high section of the lung) is plotted in a separate set of figures (upper, middle, or lower row). The lines are not a fit, but just demonstrative to connect the points. Note: The first two columns have different y-axis scales than the last column.*

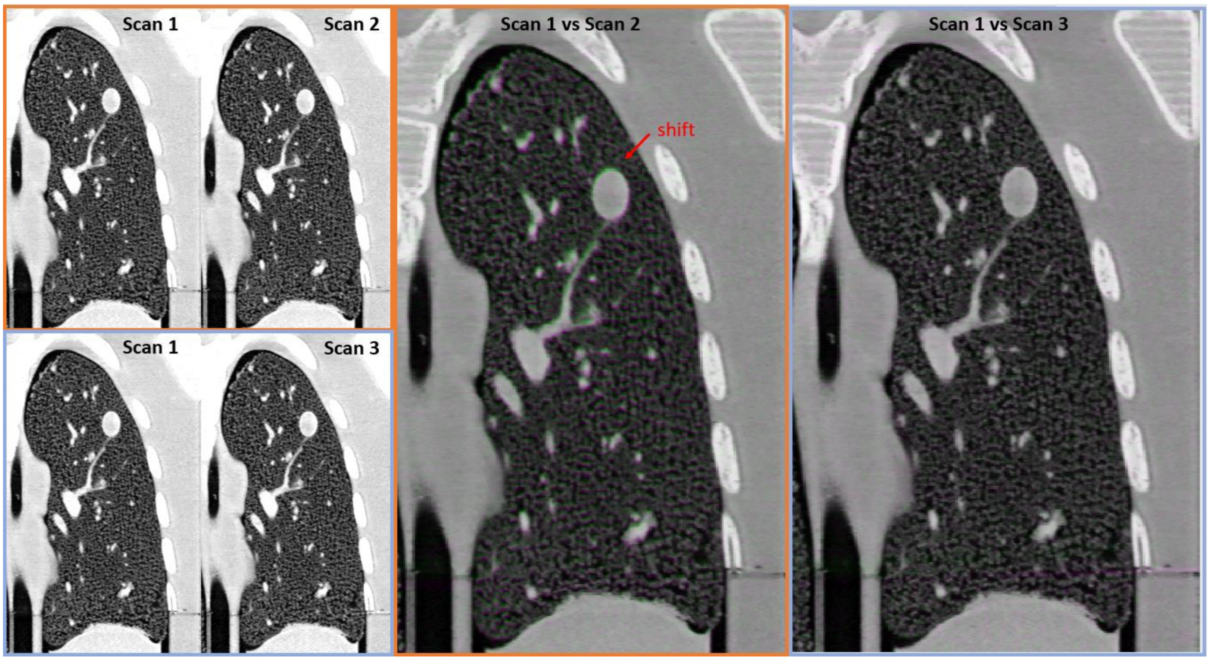

Figure S6: *Three static 3DCT images in the coronal plane of the upper lesion on the left side of the image, with scan 1 as the reference. On the right side, a comparison plot is shown to demonstrate the shift between scan 1 and scan 2 around the tumor, where green and purple indicate the difference seen between the images.*
